# Supplementary material for: Modeling glioblastoma heterogeneity as a dynamic network of cell states
Source: Mol Syst Biol. 2021 Sep 16;17(9):e10105. doi: 10.15252/msb.202010105 (PMC8444284; doi:10.15252/msb.202010105)
Supplement: Supplementary file 6 — Source Data for Figure 5 [file MSB-17-e10105-s004.zip › Figure5A_sourcedata/GSEA_3071/nosigdb_stateII.GseaPreranked.1622795151171/DALMO_BARCODENUMBER_UNPUB.html]

Details for gene set DALMO\_BARCODENUMBER\_UNPUB[GSEA]

|  || Dataset | state23071 |
| Phenotype | NoPhenotypeAvailable |
| Upregulated in class | na\_pos |
| GeneSet | DALMO\_BARCODENUMBER\_UNPUB |
| Enrichment Score (ES) | 0.5041742 |
| Normalized Enrichment Score (NES) | 3.2957523 |
| Nominal p-value | 0.0 |
| FDR q-value | 0.0 |
| FWER p-Value | 0.0 |
Table: GSEA Results Summary

  

Fig 1: Enrichment plot: DALMO\_BARCODENUMBER\_UNPUB      
 Profile of the Running ES Score & Positions of GeneSet Members on the Rank Ordered List

  

| PROBE | GENE SYMBOL | GENE\_TITLE | RANK IN GENE LIST | RANK METRIC SCORE | RUNNING ES | CORE ENRICHMENT || 1 | HIST1H4C |  |  | 2 | 1.902 | 0.0160 | Yes |
| 2 | RRM2 |  |  | 3 | 1.857 | 0.0321 | Yes |
| 3 | ANLN |  |  | 4 | 1.760 | 0.0473 | Yes |
| 4 | HIST1H1B |  |  | 7 | 1.669 | 0.0613 | Yes |
| 5 | PCLAF |  |  | 10 | 1.638 | 0.0749 | Yes |
| 6 | CDK1 |  |  | 14 | 1.540 | 0.0875 | Yes |
| 7 | DIAPH3 |  |  | 15 | 1.531 | 0.1008 | Yes |
| 8 | HJURP |  |  | 17 | 1.490 | 0.1134 | Yes |
| 9 | UBE2C |  |  | 23 | 1.438 | 0.1246 | Yes |
| 10 | CDCA5 |  |  | 41 | 1.289 | 0.1315 | Yes |
| 11 | TK1 |  |  | 42 | 1.281 | 0.1426 | Yes |
| 12 | AURKB |  |  | 44 | 1.276 | 0.1534 | Yes |
| 13 | CCNA2 |  |  | 53 | 1.230 | 0.1620 | Yes |
| 14 | SKA3 |  |  | 57 | 1.215 | 0.1718 | Yes |
| 15 | MAD2L1 |  |  | 61 | 1.199 | 0.1814 | Yes |
| 16 | UBE2T |  |  | 62 | 1.192 | 0.1918 | Yes |
| 17 | SPC25 |  |  | 68 | 1.156 | 0.2005 | Yes |
| 18 | TOP2A |  |  | 72 | 1.132 | 0.2096 | Yes |
| 19 | KIF2C |  |  | 73 | 1.132 | 0.2194 | Yes |
| 20 | KIFC1 |  |  | 88 | 1.094 | 0.2253 | Yes |
| 21 | LRR1 |  |  | 98 | 1.060 | 0.2322 | Yes |
| 22 | NCAPG |  |  | 100 | 1.056 | 0.2411 | Yes |
| 23 | DHFR |  |  | 101 | 1.051 | 0.2502 | Yes |
| 24 | MKI67 |  |  | 107 | 1.031 | 0.2579 | Yes |
| 25 | TPX2 |  |  | 111 | 1.025 | 0.2660 | Yes |
| 26 | KIF11 |  |  | 115 | 1.014 | 0.2740 | Yes |
| 27 | NCAPH |  |  | 116 | 1.013 | 0.2828 | Yes |
| 28 | SMC2 |  |  | 123 | 1.005 | 0.2900 | Yes |
| 29 | NUF2 |  |  | 125 | 1.004 | 0.2984 | Yes |
| 30 | CKS1B |  |  | 128 | 0.999 | 0.3066 | Yes |
| 31 | TACC3 |  |  | 130 | 0.992 | 0.3149 | Yes |
| 32 | ESCO2 |  |  | 144 | 0.971 | 0.3200 | Yes |
| 33 | CKAP2L |  |  | 148 | 0.959 | 0.3276 | Yes |
| 34 | NCAPG2 |  |  | 159 | 0.937 | 0.3332 | Yes |
| 35 | KIF23 |  |  | 161 | 0.936 | 0.3410 | Yes |
| 36 | GTSE1 |  |  | 167 | 0.929 | 0.3478 | Yes |
| 37 | CDCA2 |  |  | 171 | 0.922 | 0.3550 | Yes |
| 38 | CENPM |  |  | 178 | 0.901 | 0.3613 | Yes |
| 39 | SPDL1 |  |  | 199 | 0.874 | 0.3639 | Yes |
| 40 | PLK4 |  |  | 205 | 0.870 | 0.3701 | Yes |
| 41 | H2AFZ |  |  | 213 | 0.864 | 0.3758 | Yes |
| 42 | PBK |  |  | 214 | 0.864 | 0.3833 | Yes |
| 43 | CDCA3 |  |  | 219 | 0.858 | 0.3897 | Yes |
| 44 | ASPM |  |  | 231 | 0.842 | 0.3943 | Yes |
| 45 | HMGA1 |  |  | 238 | 0.834 | 0.4000 | Yes |
| 46 | RACGAP1 |  |  | 241 | 0.831 | 0.4067 | Yes |
| 47 | KIF18B |  |  | 254 | 0.815 | 0.4107 | Yes |
| 48 | TUBB4B |  |  | 271 | 0.784 | 0.4134 | Yes |
| 49 | HMGB2 |  |  | 276 | 0.780 | 0.4192 | Yes |
| 50 | CENPE |  |  | 284 | 0.775 | 0.4241 | Yes |
| 51 | DEK |  |  | 302 | 0.754 | 0.4263 | Yes |
| 52 | CENPA |  |  | 303 | 0.751 | 0.4329 | Yes |
| 53 | KIF4A |  |  | 309 | 0.746 | 0.4381 | Yes |
| 54 | CKS2 |  |  | 312 | 0.745 | 0.4440 | Yes |
| 55 | PRR11 |  |  | 314 | 0.743 | 0.4502 | Yes |
| 56 | CENPN |  |  | 320 | 0.737 | 0.4553 | Yes |
| 57 | ANP32B |  |  | 325 | 0.734 | 0.4607 | Yes |
| 58 | CENPW |  |  | 330 | 0.727 | 0.4659 | Yes |
| 59 | SPC24 |  |  | 340 | 0.719 | 0.4699 | Yes |
| 60 | ARHGAP11A |  |  | 346 | 0.712 | 0.4748 | Yes |
| 61 | HPRT1 |  |  | 351 | 0.709 | 0.4799 | Yes |
| 62 | PARPBP |  |  | 382 | 0.688 | 0.4783 | Yes |
| 63 | KIF15 |  |  | 404 | 0.676 | 0.4788 | Yes |
| 64 | SGO2 |  |  | 415 | 0.670 | 0.4821 | Yes |
| 65 | KPNA2 |  |  | 420 | 0.669 | 0.4869 | Yes |
| 66 | FOXM1 |  |  | 423 | 0.667 | 0.4922 | Yes |
| 67 | ODC1 |  |  | 430 | 0.664 | 0.4964 | Yes |
| 68 | PIF1 |  |  | 445 | 0.653 | 0.4985 | Yes |
| 69 | ACAT2 |  |  | 446 | 0.653 | 0.5042 | Yes |
| 70 | BIRC5 |  |  | 505 | 0.628 | 0.4949 | No |
| 71 | SGO1 |  |  | 528 | 0.612 | 0.4947 | No |
| 72 | MIS18BP1 |  |  | 574 | 0.588 | 0.4884 | No |
| 73 | CENPF |  |  | 575 | 0.588 | 0.4935 | No |
| 74 | CCNB1 |  |  | 579 | 0.587 | 0.4978 | No |
| 75 | SMS |  |  | 608 | 0.575 | 0.4957 | No |
| 76 | NUSAP1 |  |  | 654 | 0.550 | 0.4891 | No |
| 77 | CCT5 |  |  | 662 | 0.548 | 0.4921 | No |
| 78 | PAICS |  |  | 668 | 0.546 | 0.4955 | No |
| 79 | H2AFX |  |  | 671 | 0.544 | 0.4997 | No |
| 80 | CIP2A |  |  | 696 | 0.533 | 0.4983 | No |
| 81 | DLGAP5 |  |  | 712 | 0.529 | 0.4991 | No |
| 82 | PHF19 |  |  | 745 | 0.518 | 0.4955 | No |
| 83 | PTGES3 |  |  | 763 | 0.512 | 0.4956 | No |
| 84 | CDC20 |  |  | 844 | 0.486 | 0.4796 | No |
| 85 | LRP1 |  |  | 856 | 0.483 | 0.4810 | No |
| 86 | GADD45A |  |  | 867 | 0.480 | 0.4826 | No |
| 87 | SRSF2 |  |  | 941 | 0.461 | 0.4681 | No |
| 88 | FANCD2 |  |  | 976 | 0.450 | 0.4634 | No |
| 89 | PLK1 |  |  | 1009 | 0.442 | 0.4592 | No |
| 90 | POC1A |  |  | 1080 | 0.421 | 0.4451 | No |
| 91 | CDKN1A |  |  | 1088 | 0.419 | 0.4470 | No |
| 92 | BUB1 |  |  | 1145 | 0.408 | 0.4364 | No |
| 93 | SNRPF |  |  | 1150 | 0.407 | 0.4389 | No |
| 94 | MZT1 |  |  | 1219 | 0.393 | 0.4251 | No |
| 95 | POLD2 |  |  | 1237 | 0.389 | 0.4241 | No |
| 96 | CCT8 |  |  | 1267 | 0.384 | 0.4201 | No |
| 97 | DAZAP1 |  |  | 1330 | 0.373 | 0.4077 | No |
| 98 | ANP32E |  |  | 1334 | 0.371 | 0.4101 | No |
| 99 | ABCA5 |  |  | 1338 | 0.369 | 0.4126 | No |
| 100 | NUDT14 |  |  | 1367 | 0.363 | 0.4086 | No |
| 101 | LDHA |  |  | 1374 | 0.361 | 0.4102 | No |
| 102 | THOC3 |  |  | 1485 | 0.342 | 0.3854 | No |
| 103 | PCMT1 |  |  | 1582 | 0.327 | 0.3639 | No |
| 104 | HMGB1 |  |  | 1720 | 0.308 | 0.3319 | No |
| 105 | S100A10 |  |  | 1852 | 0.292 | 0.3013 | No |
| 106 | TAF5 |  |  | 1869 | 0.290 | 0.2998 | No |
| 107 | VPS13C |  |  | 2008 | 0.272 | 0.2672 | No |
| 108 | XRCC6 |  |  | 2180 | 0.254 | 0.2262 | No |
| 109 | TM2D1 |  |  | 2535 | -0.291 | 0.1391 | No |
| 110 | PROS1 |  |  | 2593 | -0.300 | 0.1273 | No |
| 111 | IGSF8 |  |  | 2676 | -0.312 | 0.1093 | No |
| 112 | MMP2 |  |  | 2762 | -0.328 | 0.0906 | No |
| 113 | CYSTM1 |  |  | 2791 | -0.333 | 0.0864 | No |
| 114 | YPEL3 |  |  | 2854 | -0.345 | 0.0737 | No |
| 115 | RRM2B |  |  | 2940 | -0.363 | 0.0554 | No |
| 116 | DAPL1 |  |  | 2959 | -0.366 | 0.0540 | No |
| 117 | ATP6V1G1 |  |  | 2968 | -0.367 | 0.0552 | No |
| 118 | IFI27L2 |  |  | 3071 | -0.390 | 0.0327 | No |
| 119 | KIF3C |  |  | 3087 | -0.394 | 0.0324 | No |
| 120 | PLEKHB1 |  |  | 3131 | -0.409 | 0.0250 | No |
| 121 | CHD9 |  |  | 3132 | -0.409 | 0.0286 | No |
| 122 | TMEM59L |  |  | 3300 | -0.457 | -0.0097 | No |
| 123 | LONRF2 |  |  | 3363 | -0.484 | -0.0212 | No |
| 124 | ZMAT3 |  |  | 3380 | -0.490 | -0.0210 | No |
| 125 | SEZ6L2 |  |  | 3431 | -0.513 | -0.0292 | No |
| 126 | PCDHB9 |  |  | 3682 | -0.669 | -0.0867 | No |
| 127 | IP6K2 |  |  | 3689 | -0.672 | -0.0824 | No |
| 128 | PCDH9 |  |  | 3707 | -0.691 | -0.0807 | No |
| 129 | RALGAPA2 |  |  | 3732 | -0.710 | -0.0806 | No |
| 130 | HTRA1 |  |  | 3741 | -0.719 | -0.0764 | No |
| 131 | GRIA2 |  |  | 3786 | -0.774 | -0.0808 | No |
| 132 | ST8SIA1 |  |  | 3815 | -0.801 | -0.0810 | No |
| 133 | TCEAL7 |  |  | 3819 | -0.808 | -0.0747 | No |
| 134 | CHPF |  |  | 3820 | -0.808 | -0.0677 | No |
| 135 | C9orf16 |  |  | 3833 | -0.824 | -0.0636 | No |
| 136 | PSAP |  |  | 3882 | -0.907 | -0.0679 | No |
| 137 | DDR1 |  |  | 3903 | -0.948 | -0.0648 | No |
| 138 | BTG2 |  |  | 3917 | -0.999 | -0.0594 | No |
| 139 | NEAT1 |  |  | 3976 | -1.142 | -0.0642 | No |
| 140 | PCSK1N |  |  | 3987 | -1.179 | -0.0565 | No |
| 141 | VGLL4 |  |  | 3997 | -1.227 | -0.0481 | No |
| 142 | ZBTB20 |  |  | 4024 | -1.407 | -0.0425 | No |
| 143 | ITGA7 |  |  | 4030 | -1.454 | -0.0312 | No |
| 144 | FAM210B |  |  | 4043 | -1.572 | -0.0206 | No |
| 145 | COL9A3 |  |  | 4067 | -1.892 | -0.0100 | No |
| 146 | BEX1 |  |  | 4072 | -2.034 | 0.0066 | No |
Table: GSEA details [plain text format]

  

Fig 2: DALMO\_BARCODENUMBER\_UNPUB: Random ES distribution      
 Gene set null distribution of ES for **DALMO\_BARCODENUMBER\_UNPUB**
